# Problematic Mobile Phone Use and Life Satisfaction Among University Students During the COVID-19 Pandemic in Shanghai, China

**DOI:** 10.3389/fpubh.2021.805529

**Published:** 2022-01-26

**Authors:** Wenning Jiang, Jin Luo, Hannan Guan, Feng Jiang, Yi-Lang Tang

**Affiliations:** ^1^Antai College of Economics and Management, Shanghai Jiao Tong University, Shanghai, China; ^2^School of International and Public Affairs, Shanghai Jiao Tong University, Shanghai, China; ^3^Institute of Healthy Yangtze River Delta, Shanghai Jiao Tong University, Shanghai, China; ^4^School of Education, Shanghai Jiao Tong University, Shanghai, China; ^5^Department of Education Information Technology, East China Normal University, Shanghai, China; ^6^Department of Psychiatry and Behavioral Sciences, Emory University, Atlanta, GA, United States; ^7^Atlanta Veterans Affairs Medical Center, Decatur, GA, United States

**Keywords:** mobile phone use, life satisfaction, association, university students, China

## Abstract

**Objective:**

This study examined problematic mobile phone use (PMPU) and its relationship with life satisfaction in Chinese university students during the pandemic.

**Methods:**

An anonymous online survey was conducted in a university in China. The Mobile Phone Addiction Index (MPAI) and the Satisfaction with Life Scale (SWLS) were used to assess the severity of problematic mobile phone use and life satisfaction, respectively. Data on demographic and health-related factors were also collected.

**Results:**

A total of 1,491 undergraduate students (73.3% were male) completed the survey. On average, students in the survey reported spending 7.4 ± 4.3 h/day on phone use. Their MPAI score was 38.1 ± 13.3 and SWLS score was 24.9 ± 6.8, respectively. After controlling for confounding factors, the MPAI score was significantly associated with lower life satisfaction. Multiple linear regression revealed that higher monthly allowances, frequent insomnia, longer phone use duration were significantly associated with PMPU.

**Conclusion:**

University students in China spend nearly half of their waking hours on mobile phone use, significantly longer than before the COVID-19 pandemic. PMPU is associated with insomnia, lower life satisfaction and higher allowances. If the trend continues after the pandemic, interventions may be needed. Increase in-person interactions, limiting online social and gaming time, awareness campaign may be effective in reducing the impact of PMPU and improve life satisfaction.

## Introduction

Smartphones have become a necessity and the most important communication tool because of their convenience and accessibility (1–3), and this is especially true for young people. Due to the technological advances, smartphones have been used in academic, professional, social and recreational activities, including those tasks that were previously only possible on computers. In the meantime, excessive smartphone use could lead to a series negative health outcomes, including depression, anxiety, sleep deprivation and insomnia (4–7), and low life satisfaction. Furthermore, excessive smartphone use may increase the risk of problematic mobile phone use (PMPU), and even lead to smartphone addiction (8).

Of note, neither smartphone addiction nor internet addiction is officially listed as a diagnosis in any major diagnostic systems and it is controversial to consider it as a diagnostic entity. However, a few other terms have been used to describe this phenomenon: problematic mobile phone use, mobile phone addiction, excessive mobile phone use, and compensatory mobile phone use (9). PMPU is characterized by excessive attention and uncontrolled dedication to one's cell phone use (10).

In China, young people are the largest growing group of smartphone users, especially university students (11). University students tend to routinely use smartphones in their study and other daily activities (12). In 2018, a survey demonstrated that Chinese university students spend over 5 h/day on mobile phones, and ~4/5 (79%) use smartphones in class (2).

The COVID-19 has dramatically changed people's lives in numerous ways. In the early stages of the pandemic, a range of emergency public health measures were adopted, such as universal masking, social distancing, locking down, school closure, and public transportation suspension. One adaptive behavioral change during the extraordinary times is increased use of the internet and smartphone for either professional use or personal use. One survey found that, during the COVID-19 pandemic, adolescents spent more time on the internet to study, play games, and chat with friends (13). This large-scale social isolation and overexposure to the mobile devices may contribute to a few mental health issues, including the potential to have PMPU.

As an important element in happiness, life satisfaction is the gap between what people have and what they want (14), which correlates positively with academic performance and productivity (15). It is believed that life satisfaction is affected by conditions such as health, socioeconomic status, and activities (16).

To date, although a few studies have examined internet use and smartphone use in adolescents, middle and high school students (17–22), few studies examined the smartphone use pattern and PMPU among university students in China during the COVID-19 pandemic. Furthermore, no studies have examined the association between PMPU and life satisfaction using standard structured instruments. Therefore, we designed this study and collected data among university students during the COVID-19 pandemic. We firstly surveyed the average time spent on smartphones, then examined the factors associated with PMPU, with focus on the relationship between PMPU and life satisfaction.

## Materials and Methods

### Study Design and Participants

This cross-sectional study was conducted in a comprehensive university from July 7 to 17 in 2021. Before the start of the survey, we calculated the minimum sample size using the following formula:


$$\begin{array}{l} \text{n}=\frac{\text{N}}{1+\frac{4d^{2}\left(N-\;1\right)}{z_{\alpha }^{2}}} \end{array}$$

where n is the minimum sample size, N the size of the students in Shanghai Jiao Tong University, d the maximum error of estimate, and z_α_ the critical value of normal distribution at the assumed confidence level.

Three schools (Antai College of Economics & Management; School of International and Public Affairs; School of Electronic, Information and Electrical Engineering) were selected as convenience samples, while all full-time undergraduates in the selected schools were invited to participate. The weblink of the study was posted via WeChat, a popular social app in mainland China.

The Ethics Committee in Shanghai Jiao Tong University approved the study protocol (approval number: H2021158I). Each participant provided written informed consent before they responded to the questionnaire.

### Measures

#### Socio-Demographic Characteristics

We collected socio-demographic data of the participants, including gender, age, grade, place of hometown, ethnicity, major, monthly allowances, and relationship status. We also collected information on alcohol use, cigarette use, and insomnia based on literature review (2).

#### PMPU

PMPU was assessed using the Mobile Phone Addiction Index (MPAI), which has been widely used in various studies (23, 24). MAPI was developed by Leung to rate the comprehensive level of mobile phone addiction and related symptoms, including the inability to control craving, feel anxious and lost, withdrawal or escape, and productivity loss (25). All 17 items were rated on a 5-point Likert-type scale, ranging from 1 (Never) to 5 (Always), while higher total scores indicate higher levels of mobile phone addiction (23). The Cronbach's α of MPAI was 0.93 in the present study.

#### Life Satisfaction

Participants' overall life satisfaction was assessed using the Satisfaction with Life Scale (SWLS) (14), which has been widely used around the world with good reliability and validity (26, 27). The scale assesses an individual's satisfaction with life as a whole. It has 5 items, such as “The conditions of my life are excellent.” Participants rated the items on a 5-point Likert scale, ranging from 1 (Strongly Disagree) to 5 (Strongly Agree), with higher scores indicative of better-perceived life satisfaction. The Cronbach's α of SWLS in our samples was 0.94.

### Data Analysis

One-sample K-S test was used to examine the normality of the data. Descriptive analyses for the sample's socio-demographic, PMPU, life satisfaction, and other related factors were conducted. The associations between PMPU, life satisfaction, and other factors were examined with independent samples *t*-test, analysis of variance (ANOVA), chi-square test, and Pearson correlation analysis, as appropriate. The independent associations between PMPU and life satisfaction were determined with a stepwise multiple linear regression after controlling for significant correlates as identified in the univariate analyses. The independent factors associated with PMPU were identified through a stepwise method of multiple linear regression; MPAI score was entered as the dependent variable, while its significant correlates in univariate analyses were involved as the independent variables. Data analyses were carried out with the STATA software version 16.0 (Stata Corporation, College Station, TX, USA), with the significance level at the *p*-value of 0.05 (two-tailed).

## Results

In total, 4,561 undergraduates were invited to participate and 1,534 students responded (response rate of 33.63%). The relatively low response rate might be attributable to the busy schedules at the end of the Spring semester. Therefore, 1,491 undergraduates completed the survey without logical errors and were included in the statistical analysis.

Their mean age was 20.8 ± 2.9 years, and 73.3% were male. They spent 7.4 ± 4.3 h/day on smartphones, with the MPAI score of 38.1 ± 13.3. Their SWLS score was 24.9 ± 6.8. Table 1 shows the detailed information of their social-demographic and related characteristics.

**Table 1:** Characteristics of 1,491 participants.

**Characteristic**	***N***	**%**
**Gender**		
Male	1,093	73.31
Female	398	26.69
**Grade**		
Freshman	401	26.89
Sophomore	404	27.10
Junior	457	30.65
Senior	229	15.36
**Hometown setting**		
Urban	979	65.66
Rural	512	34.34
**Ethnicity**		
Han Chinese	1,376	92.29
Minority nationality	115	7.71
**Major**		
Engineering	494	33.13
Science	464	31.12
Economy	108	7.24
Others	425	28.50
**Monthly allowances (RMB)**		
<1,000	184	12.34
1,000–1,499	509	34.14
1,500–1,999	391	26.22
2,000–2,499	228	15.29
2,500–2,999	65	4.36
≥3,000	114	7.65
**Relationship status**		
Not dating nor married	864	57.95
Dating but unmarried	534	35.81
Married	54	3.62
Others	39	2.62
**Insomnia**		
No	623	41.78
Seldom ( ≤ 3 times/month)	450	30.18
Sometimes (1–2 times/week)	275	18.44
Often (3–5 times/week)	109	7.31
Daily (>5 times/week)	34	2.28
**Cigarette use**		
No	1,177	78.94
Ex-smoker	185	12.41
Current smoker	129	8.65
**Alcohol use**		
Never	862	57.81
Rare ( ≤ 2 times/month)	410	27.50
Sometimes ( ≤ 4 times/month)	145	9.73
Often ( ≤ 12 times/month)	47	3.15
Always (>12 times/month)	27	1.81
	**Mean**	**SD**
Age (years)	20.83	2.89
MAPI	38.12	13.33
SWLS	24.87	6.81
Phone use duration (hours)	7.39	4.32

*MAPI, mobile phone addiction index; SWLS, the satisfaction with life scale*.

Table 2 displays the results of univariate analyses, showing the factors associated with PMPU and life satisfaction.

**Table 2:** Univariate analyses of factors associated with PMPU and SWLS.

**Variable**	**MPAI score**			**SWLS**		
	**Mean ±SD**	***t*/F**	***P***	**Mean ±SD**	***t*/F**	***P***
Gender		−0.60	0.549		3.29	**0.001**
Male	38.00 ± 13.41			25.22 ± 6.84		
Female	38.46 ± 13.13			23.91 ± 6.67		
Grade		1.49	0.214		1.23	0.297
Freshman	37.14 ± 13.53			24.37 ± 7.04		
Sophomore	37.89 ± 13.54			24.86 ± 6.97		
Junior	38.65 ± 12.92			25.11 ± 6.42		
Senior	39.18 ± 13.38			25.31 ± 6.89		
Place of hometown		0.01	0.916		0.79	0.430
Urban	38.09 ± 13.52			24.97 ± 6.86		
Rural	38.17 ± 12.98			24.68 ± 6.74		
Ethnic groups		−2.28	**0.022**		0.73	0.465
Han	37.89 ± 13.29			24.91 ± 6.81		
Minority nationality	40.84 ± 13.65			24.43 ± 6.89		
Specialty		4.65	**0.003**		7.08	**<0.001**
Engineering	37.87 ± 12.49			24.10 ± 6.32		
Science	36.74 ± 14.21			25.87 ± 7.22		
Economy	37.72 ± 12.26			23.48 ± 6.32		
Others	40.02 ± 13.38			25.04 ± 6.89		
Monthly allowances (RMB)		6.19	**<0.001**		1.57	0.166
<1,000	34.34 ± 14.55			24.90 ± 8.56		
1,000–1,499	37.56 ± 12.70			24.45 ± 6.26		
1,500–1,999	38.06 ± 12.60			24.67 ± 6.46		
2,000–2,499	41.05 ± 13.63			25.54 ± 6.71		
2,500–2,999	40.37 ± 11.71			24.92 ± 6.58		
≥3,000	39.82 ± 15.17			26.04 ± 7.44		
Relationship status		8.12	**<0.001**		6.40	**<0.001**
Not dating nor married	36.78 ± 13.39			24.34 ± 6.75		
Dating but unmarried	39.58 ± 12.53			25.31 ± 6.68		
Married	42.48 ± 16.25			27.31 ± 7.71		
Others	41.87 ± 14.62			27.21 ± 7.49		
Insomnia		37.96	**<0.001**		11.47	**<0.001**
No	33.90 ± 13.17			26.17 ± 6.85		
Seldom ( ≤ 3 times/month)	39.22 ± 12.03			24.37 ± 6.39		
Sometimes (1–2 times/week)	41.80 ± 12.26			23.85 ± 6.10		
Often (3–5 times/week)	45.19 ± 12.74			22.78 ± 7.51		
Daily (>5 times/week)	48.59 ± 16.36			22.76 ± 10.02		
Cigarette use		17.86	**<0.001**		1.11	0.331
No	37.11 ± 13.16			24.83 ± 6.71		
Ex-smoker	42.99 ± 12.15			25.48 ± 6.87		
Current smoker	40.33 ± 14.72			24.37 ± 7.67		
Alcohol use		10.63	**<0.001**		4.18	**0.002**
Never	36.34 ± 13.51			25.42 ± 6.86		
Rare ( ≤ 2 times/month)	39.89 ± 12.47			23.81 ± 6.41		
Sometimes ( ≤ 4 times/month)	42.34 ± 11.92			25.01 ± 6.54		
Often ( ≤ 12 times/month)	42.19 ± 13.50			23.83 ± 7.61		
Always (>12 times/month)	38.48 ± 17.32			24.63 ± 9.37		

*Bold value for p < 0.05*.

Table 3 shows the Pearson correlation coefficients of PMPU and life satisfaction with age and smartphone use time. Phone use time was significantly associated with MPAI score (*r* = 0.135, *p* < 0.05).

**Table 3:** Pearson correlation analysis for PMPU and SWLS.

**Variable**	**MPAI**	**SWLS**
Age (years)	0.015	0.015
Phone use duration	0.135*	−0.015

*p < 0.05.

After controlling for other related factors, MPAI score was significantly associated with lower life satisfaction (β = −0.10, *p* < 0.001) (Table 4).

**Table 4:** Association of PMPU and SWLS.

**Variable**	**β**	**95% CI (Lower)**	**95% CI (Upper)**	***P***
MAPI	−0.10	−0.13	−0.07	**<0.001**
Female	−1.15	−1.90	−0.40	**0.003**
**Marriage**				
Dating but unmarried	1.43	0.72	2.13	**<0.001**
Married	4.21	2.38	6.04	**<0.001**
Others	3.64	1.54	5.75	**0.001**
**Insomnia**				
Seldom	−1.28	−2.09	−0.48	**0.002**
Sometimes	−1.87	−2.82	−0.92	**<0.001**
Often	−2.85	−4.22	−1.48	**<0.001**
Daily	−2.67	−4.96	−0.37	**0.023**

*Bold value for p < 0.05*.

We also found that PMPU was significantly associated with higher monthly allowances, frequent insomnia, longer phone use time (Table 5).

**Table 5:** Independent correlates of PMPU.

**Variable**	**β**	**95% CI (Lower)**	**95% CI (Upper)**	***P***
**Monthly allowances (RMB)**				
1,000–1,499	3.52	1.39	5.66	**0.001**
1,500–1,999	3.24	1.02	5.46	**0.004**
2,000–2,499	5.85	3.38	8.31	**<0.001**
2,500–2,999	5.00	1.98	8.03	**0.006**
≥3,000	5.02	1.45	8.59	**0.002**
**Insomnia**				
Seldom ( ≤ 3 times/month)	5.00	3.47	6.54	**<0.001**
Sometimes (1–2 times/week)	7.47	5.67	9.27	**<0.001**
Often (3–5 times/week)	10.28	7.68	12.87	**<0.001**
Daily	14.01	9.61	18.42	**<0.001**
Phone use duration	0.28	0.12	0.43	**<0.001**

*Bold value for p < 0.05*.

## Discussion

Based on a large (>1,000), relatively homogenous sample of undergraduate students from a university, we found that students spent 7.4 ± 4.3 h/day on phone use. Their MPAI score was 38.12 ± 13.33 and SWLS score was 24.987 ± 6.81, respectively. We also found the MPAI score was significantly associated with lower life satisfaction. Multiple linear regression revealed that PMPU was significantly associated with higher monthly allowances, frequent insomnia, and longer phone use duration in this sample.

To our best knowledge, this survey was one of the first that examined the relationship between PMPU and life satisfaction among Chinese university students during the COVID-19 pandemic. This study found a negative association between excessive mobile phone use and life satisfaction, suggesting a link between PMPU and lower life satisfaction. While the mechanism and causality are unclear, some studies reported significant association between excessive mobile phone use and poor sleep quality, insomnia (28–30), depression and anxiety (6, 31), all of which may be linked to poor life satisfaction or quality of life (QOL) (32–34). Similar findings between PMPU and life satisfaction have been reported by other studies. Reports from the United States and Lebanon also found that PMPU was negatively associated with life satisfaction, mediated by academic performance and stress (35, 36). Another study of Chinese university students demonstrated that the severity of mobile phone addiction was significantly associated with lower scores on all domains of QOL measures (2).

In the current study, we found the average duration of mobile phone use was 7.39 h, which was much longer than previous studies, especially those prior to the COVID-19 pandemic. A study conducted in December 2018 in Shenzhen, a city in south China, showed that youth (18–24 years old) people used mobile phones for 3.78 ± 2.51 h per day before the pandemic (37). Xie et al. showed that male university students in pre-pandemic Macau used mobile phones 2.7 ± 2.4 h per day, while female students 3.0 ± 2.5 h (38). Another study in Turkey Inonu University before the COVID-19 pandemic found that 21.6% of students used cell phones for 3 h or less, 31.7% between 4 and 5 h, 18.5% between 6 and 7 h, and 28.2% longer than 8 h (39). The survey of Mobile phone usage in 2018 found university students in China on average spent over 5 h/day on mobile phones, which is much closer to our findings (2). The significant difference in mobile phone use among different studies may be due to different samples, time of survey and how the questions were asked. During the COVID-19 period, university students needed to use smartphones for academic activities, including online class and learning, which may explain the longer phone use (40), although our data did not differentiate between personal use and professional use.

An interesting finding of our study is that the MAPI score was lower than in several previous studies. For example, Liu et al. found that the MPAI score in male medical college students in Shanghai was 44.94 ± 12.08, female 45.25 ± 11.87 (41). A study of high school students in Sichuan and Chongqing showed a little higher MPAI score with a mean of 41.65 than our study (24). Again the sampling methods, samples' demographic features may explain some of the difference. It is also possible that although our sample reported longer time on smartphone, they had used it more academic activities instead of smartphone gaming, therefore less distress and impairments were experienced, as other types of smartphone activities such as internet gaming were associated with psychological distress (42, 43).

In the regression model, we found that PMPU was significantly associated with students' monthly allowances, which often reflect their family socioeconomic status. This finding is in line with findings of a study among Iranian medical university students, which demonstrated that family economic status was a significant predictor of mobile phone dependency (44). The exact mechanism between socioeconomic status and PMPU warrants further investigations.

Several limitations about this study need to be noted. First, due to the nature of a cross-sectional survey, a causal relationship between PMPU and other variables could not be examined. Second, some important information related to PMPU and life satisfaction, such as personality and academic performance, were not recorded. Third, we did not specify whether the time spent on smartphone was for academic activity, social communication or gaming, which may provide more helpful information regarding intervention. Fourth, as the instruments were self-reported, the recall bias and response bias cannot be ruled out in the study. Finally, as the participating schools were selected by convenience, this could lead to sampling bias, therefore the generalizability of the conclusions may be limited.

## Conclusions

In conclusion, we found university undergraduate students in China spend nearly half of their waking hours on mobile phone use, significantly longer than before the COVID-19 pandemic. PMPU is associated with insomnia, lower life satisfaction and higher allowances. Awareness campaign is needed. If this trend continues, interventions may be indicated, including facilitating in person interactions, limiting online social and gaming time. These changes may be effective in reducing the impact of PMPU and improve life satisfaction among students.

## Data Availability Statement

The raw data supporting the conclusions of this article will be made available by the authors, without undue reservation.

## Ethics Statement

The studies involving human participants were reviewed and approved by the Ethics Committee in Shanghai Jiao Tong University. The patients/participants provided their written informed consent to participate in this study.

## Author Contributions

FJ and Y-LT made substantial contributions to the study design. JL, WJ, and HG collected data. FJ analyzed the data. JL and FJ interpreted the results of analysis and completed the manuscripts. Y-LT critically revised the manuscript. All authors contributed to the article and approved the submitted version.

## Funding

This work was supported by the Shanghai Philosophy and Social Science Planning Project (No.2020EGL001).

## Conflict of Interest

The authors declare that the research was conducted in the absence of any commercial or financial relationships that could be construed as a potential conflict of interest.

## Publisher's Note

All claims expressed in this article are solely those of the authors and do not necessarily represent those of their affiliated organizations, or those of the publisher, the editors and the reviewers. Any product that may be evaluated in this article, or claim that may be made by its manufacturer, is not guaranteed or endorsed by the publisher.
